# Supplementary material for: Supercoil Levels in E. coli and Salmonella Chromosomes Are Regulated by the C-Terminal 35–38 Amino Acids of GyrA
Source: Microorganisms. 2019 Mar 15;7(3):81. doi: 10.3390/microorganisms7030081 (PMC6463007; doi:10.3390/microorganisms7030081)
Supplement: Supplementary file 1 [file microorganisms-07-00081-s001.pdf]

Supplementary DATA

Fig. S1. The Supercoil Sensor and mechanism of the $\gamma\delta$ resolution reaction. A. The 9 kb supercoil sensor is shown inserted at a Frt site upstream of the highly transcribed *Salmonella* ATP operon (the first two genes *atpI* and *atpB*). The sensor includes a complete copy of the lac operon, a gentamycin resistance gene (Gn) shown in green, two directly repeated Res sites (red) flanked by two Frt sites (blue). B. Recombination in the Tn3/$\gamma\delta$ resolvase pathway requires two 114 bp sites (Res) with binding sites for three dimers of the resolvase. The sites are ResI (blue), ResII (red), and ResIII (yellow). C. Supercoil diffusion is required to form an active synapse in which two directly repeated sites entrap 3 negative crossing nodes. Only resolvase dimers bound to Res site I, (blue box and blue oval) can catalyze strand exchange. Movement of the interwound DNA strands promotes formation of the three-node synapse by reversible branching and slithering. Recombination generates an irreversible strand exchange with two supercoiled molecules singly linked as catenanes. D. The dependence of – supercoiling for plasmids recombination *in vitro* is shown by the scale on the left [1]. The inferred diffusible supercoil density for recombination of a 9 Kb interval in the *Salmonella* chromosome in vivo is shown on the bottom [2].

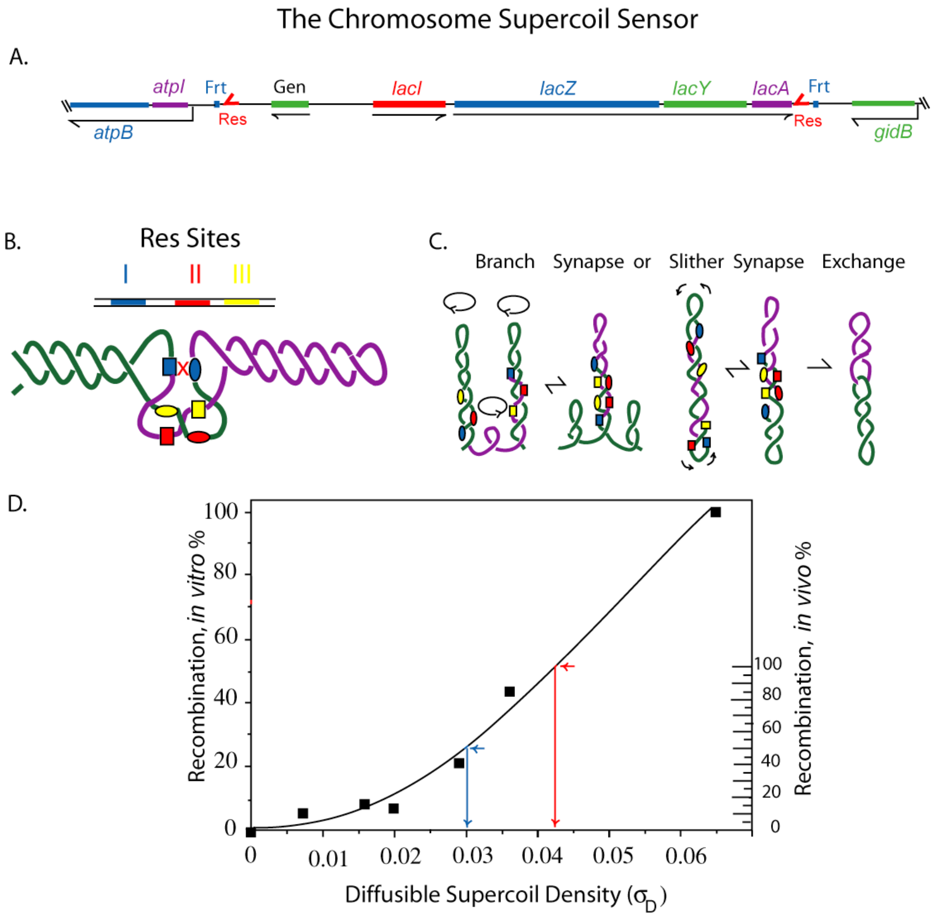

Table S1.

Strains Used

Strain	Genotype	Plasmid	Cs
NH2002	LT2 WT		
NH6000	*atpI* < Frt Res lac Gn Res Frt > *gidB*	pJBRES *30’*	85
NH6001	*STM3261* < Frt Res *lac* Gn Res Frt > *STM3262*	pJBRES *30’*	71
NH6002	*smpB* < Frt Res *lac* Gn Res Frt > *STM2689*	pJBRES *30’*	58
NH6003	*STM2135* < Frt Res *lac* Gn Res Frt > *yegQ*	pJBRES *30’*	45
NH6005	*STM1554* < Frt Res *lac* Gn Res Frt > *STM1553*	pJBRES *30’*	33
NH6006	*STM0951* < Frt Res *lac* Gn Res Frt > *STM0952*	pJBRES *30’*	21
NH6007	*ampH* < Frt Res *lac* Gn Res Frt > *sbmA*	pJBRES *30’*	9
NH6008	*STM4442*::< Frt Res *lac* Gn Res Frt >	pJBRES *30’*	96
NH6009	*marC*::< Frt Res *lac* Gn Res Frt >	pJBRES *30’*	33
NH6010	*STM1612*::< Frt Res *lac* Gn Res Frt >	pJBRES *30’*	35
NH6011	*ycjG*:: < Frt Res *lac* Gn Res Frt >	pJBRES *30’*	36
NH6012	*acnA* < Frt Res *lac* Gn Res Frt > *cysB*	pJBRES *30’*	37
NH6016	NH6000 *zeh754*::Tn*10 gyrA213*	pJBRES *30’*	85
NH6018	NH6005 *zeh754*::Tn*10 gyrA213*	pJBRES *30’*	85
NH6019	NH6000 *zeh754*::Tn*10 gyrA209*	pJBRES *30’*	85
NH6020	NH6001 *zeh754*::Tn*10 gyrA209*	pJBRES *30’*	71
NH6021	NH6002 *zeh754*::Tn*10 gyrA209*	pJBRES *30’*	58
NH6022	NH6003 *zeh754*::Tn*10 gyrA209*	pJBRES *30’*	45
NH6024	NH6005 *zeh754*::Tn*10 gyrA209*	pJBRES *30’*	33
NH6025	NH6006 *zeh754*::Tn*10 gyrA209*	pJBRES *30’*	21
NH6026	NH6007 *zeh754*::Tn*10 gyrA209*	pJBRES *30’*	9
NH6027	NH6008 *zeh754*::Tn*10 gyrA209*	pJBRES *30’*	96
NH6028	NH6000 *zib6794*::Tn*10 gyrB652*	pJBRES *30’*	85
NH6029	NH6001 *zib6794*::Tn*10 gyrB652*	pJBRES *30’*	71
NH6030	NH6002 *zib6794*::Tn*10 gyrB652*	pJBRES *30’*	58
NH6031	NH6003 *zib6794*::Tn*10 gyrB652*	pJBRES *30’*	45
NH6033	NH6005 *zib6794*::Tn*10 gyrB652*	pJBRES *30’*	33
NH6034	NH6006 *zib6794*::Tn*10 gyrB652*	pJBRES *30’*	21
NH6035	NH6007 *zib6794*::Tn*10 gyrB652*	pJBRES *30’*	9
NH6036	NH6008 *zib6794*::Tn*10 gyrB652*	pJBRES *30’*	96
NH6037	NH6000 *zib748*::Tn*10 gyrB1820*	pJBRES *30’*	85
NH6040	MH6000 *zgc2393*::Tn*10 parC281*	pJBRES *30’*	85
NH6043	NH6000 *zgc2393*::Tn*10 parE206*	pJBRES *30’*	85

NH6044	NH6001 *zgc2393*::Tn*10 parE206*	pJBRES *30’*	71
NH6045	NH6002 *zgc2393*::Tn*10 parE206*	pJBRES *30’*	58
NH6046	NH6003 *zgc2393*::Tn*10 parE206*	pJBRES *30’*	45
NH6048	NH6005 *zgc2393*::Tn*10 parE206*	pJBRES *30’*	33
NH6049	NH6006 *zgc2393*::Tn*10 parE206*	pJBRES *30’*	21
NH6056	NH6007 *zgc2393*::Tn*10 parE206*	pJBRES *30’*	9
NH6058	NH6008 *zgc2393*::Tn*10 parE206*	pJBRES *30’*	96
NH6072	STM2655:: < Frt Res *lac* Gn Res Frt >	pJBRES *30’*	57.64
NH6073	*clpB* < Frt Res *lac* Gn Res Frt > *rrlG*	pJBRES *30’*	57.65
NH6108	NH6001 *zib748*::Tn*10 gyrB1820*	pJBRES *30’*	71
NH6109	NH6002 *zib748*::Tn*10 gyrB1820*	pJBRES *30’*	58
NH6110	NH6003 *zib748*::Tn*10 gyrB1820*	pJBRES *30’*	45
NH6111	NH6005 *zib748*::Tn*10 gyrB1820*	pJBRES *30’*	33
NH6112	NH6006 *zib748*::Tn*10 gyrB1820*	pJBRES *30’*	21
NH6113	NH6007 *zib748*::Tn*10 gyrB1820*	pJBRES *30’*	9
NH6114	NH6008 *zib748*::Tn*10 gyrB1820*	pJBRES *30’*	96
NH6118	NH6009 *zib748*::Tn*10 gyrB1820*	pJBRES *30’*	33’
NH6119	NH6010 *zib748*::Tn*10 gyrB1820*	pJBRES *30’*	35
NH6120	NH6011 *zib748*::Tn*10 gyrB1820*	pJBRES *30’*	36
NH6121	NH6012 *zib748*::Tn*10 gyrB1820*	pJBRES *30’*	37
NH6222	NH6000 *rpoC* (β' Δ215-220)	pJBRES *30’*	85
NH6223	NH6001 *rpoC* (β' Δ215-220)	pJBRES *30’*	71
NH6224	NH6002 *rpoC* (β' Δ215-220)	pJBRES *30’*	58
NH6225	NH6003 *rpoC* (β' Δ215-220)	pJBRES *30’*	45
NH6226	NH6005 *rpoC* (β' Δ215-220)	pJBRES *30’*	33
NH6227	NH6006 *rpoC* (β' Δ215-220)	pJBRES *30’*	21
NH6228	NH6007 *rpoC* (β' Δ215-220)	pJBRES *30’*	9
NH6229	NH6008 *rpoC* (β' Δ215-220)	pJBRES *30’*	96
NH6230	NH6073 *rpoC* (β' Δ215-220)	pJBRES *30’*	57.65
NH6231	NH6072 *rpoC* (β' Δ215-220)	pJBRES *30’*	57.64

List and genetic structure of all strains used in this study. All strains were created for this or previous studies related to this work.

Table S2. Resolution and σ $_D$ values of WT and chimeric strains carrying *gyrA*, *gyrB* or both genes from *E. coli*.

Strain	Map Position	Relevant Subunits	Resolution Efficiency	MIF	Apparent σ $_D$	Average σ $_D$ (w/o Cs 33)
NH6259	Cs 85	$A_{St}B_{St}$	66 ± 8%	1	-0.034	
NH6260	Cs 71	$A_{St}B_{St}$	62 ± 10%	1	-0.033	
NH6261	Cs 58	$A_{St}B_{St}$	69 ± 7%	1	-0.035	
NH6257	Cs 57.65	$A_{St}B_{St}$	69 ± 11%	1	-0.035	
NH6258	Cs 57.64	$A_{St}B_{St}$	31 ± 11%	1	-0.024	
NH6262	Cs 45	$A_{St}B_{St}$	69 ± 12%	1	-0.035	
NH6263	Cs 33	$A_{St}B_{St}$	34 ± 9%	1	-0.025	
NH6264	Cs 21	$A_{St}B_{St}$	66 ± 11%	1	-0.034	
NH6265	Cs 9	$A_{St}B_{St}$	49 ± 17%	1	-0.030	*WT* mean
NH6266	Cs 96	$A_{St}B_{St}$	66 ± 15%	1	-0.034	- 0.032±0.004
NH6273	Cs 85	$A_{St}B_{Ec}$	78 ± 1%	0.8	- 0.037	
NH6274	Cs 71	$A_{St}B_{Ec}$	63 ± 9%	1.0	-0.034	
NH6275	Cs 58	$A_{St}B_{Ec}$	66 ± 7%	1.0	- 0.034	
NH6271	Cs 57.65	$A_{St}B_{Ec}$	89 ± 3%	0.8	-0.040	
NH6272	Cs 57.64	$A_{St}B_{Ec}$	50 ± 7%	0.6	-0.030	
NH6276	Cs 45	$A_{St}B_{Ec}$	72 ± 7%	1.0	- 0.036	
NH6277	Cs 33	$A_{St}B_{Ec}$	13 ± 3%	2.6	- 0.016	
NH6278	Cs 21	$A_{St}B_{Ec}$	54 ± 1%	1.2	- 0.031	
NH6279	Cs 9	$A_{St}B_{Ec}$	29 ± 8%	1.7	- 0.023	$A_{St}B_{Ec}$ mean
NH6280	Cs 96	$A_{St}B_{Ec}$	51 ± 2%	1.3	- 0.030	- 0.031±0.007
NH6284	Cs 85	$A_{Ec}B_{St}$	30 ± 10%	2.2	- 0.024	
NH6285	Cs 71	$A_{Ec}B_{St}$	21 ± 2%	3.0	- 0.020	
NH6286	Cs 58	$A_{Ec}B_{St}$	26 ± 3%	2.7	- 0.022	
NH6282	Cs 57.65	$A_{Ec}B_{St}$	10 ±1%	6.9	-0.014	
NH6283	Cs 57.64	$A_{Ec}B_{St}$	10 ± 13%	3.1	-0.014	
NH6287	Cs 45	$A_{Ec}B_{St}$	29 ± 8%	2.4	- 0.023	
NH6288	Cs 33	$A_{Ec}B_{St}$	11 ± 3%	3.1	- 0.015	
NH6289	Cs 21	$A_{Ec}B_{St}$	48 ±7%	1.4	- 0.030	
NH6290	Cs 9	$A_{Ec}B_{St}$	54 ± 15%	0.9	- 0.031	$A_{Ec}B_{St}$ mean
NH6291	Cs 96	$A_{Ec}B_{St}$	31 ± 12%	2.1	- 0.024	-0.022±0.006
NH6295	Cs 85	$A_{Ec}B_{Ec}$	41 ± 10%	1.6	- 0.027	
NH6296	Cs 71	$A_{Ec}B_{Ec}$	30 ± 3%	2.1	- 0.024	
NH6297	Cs 58	$A_{Ec}B_{Ec}$	51 ± 5%	1.4	- 0.030	
NH6293	Cs 57.65	$A_{Ec}B_{Ec}$	31 ± 7%	2.2	-0.024	
NH6294	Cs 57.64	$A_{Ec}B_{Ec}$	6 ± 5%	5.2	-0.011	
NH6298	Cs 45	$A_{Ec}B_{Ec}$	47 ± 4%	1.5	- 0.029	
NH6299	Cs 33	$A_{Ec}B_{Ec}$	12 ± 1%	2.8	- 0.015	
NH6300	Cs 21	$A_{Ec}B_{Ec}$	29 ± 3%	2.3	- 0.023	
NH6301	Cs 9	$A_{Ec}B_{Ec}$	14 ± 4%	3.5	- 0.016	$A_{Ec}B_{Ec}$ mean
NH6302	Cs 96	$A_{Ec}B_{Ec}$	31 ± 2%	2.1	- 0.024	-0.022±0.006

Table S3. Results of resolution measurement and apparent σ_D values of strains with GyrA chimeras containing *E. coli* GyrA with either a half ($A_{Ec<1/2\ CTD\text{-}ST>}$) or whole ($A_{Ec<CTD\text{-}ST>}$) CTD domain from *S. typhimurium* and *gyrB* from either *E. coli* or *S. typhimurium*.

Strain	Map Position	Relevant Subunits	Resolution Efficiency	MIF	Apparent σ_D	Average σ_D (w/o Cs 33)
NH6356	Cs 85	$A_{Ec<1/2\ CTD\text{-}ST>}B_{St}$	81±1%	0.8	-0.038	
NH6357	Cs 71	$A_{Ec<1/2\ CTD\text{-}ST>}B_{St}$	71±2%	0.9	-0.036	
NH6358	Cs 58	$A_{Ec<1/2\ CTD\text{-}ST>}B_{St}$	73±1%	0.9	-0.036	
NH6354	Cs 57.65	$A_{Ec<1/2\ CTD\text{-}ST>}B_{St}$	71±2%	1.0	-0.036	
NH6355	Cs 57.64	$A_{Ec<1/2\ CTD\text{-}ST>}B_{St}$	39±1%	0.8	-0.028	
NH6359	Cs 45	$A_{Ec<1/2\ CTD\text{-}ST>}B_{St}$	71±2%	1.0	-0.036	
NH6360	Cs 33	$A_{Ec<1/2\ CTD\text{-}ST>}B_{St}$	35±1%	1.0	-0.025	
NH6361	Cs 21	$A_{Ec<1/2\ CTD\text{-}ST>}B_{St}$	67±1%	1.0	-0.035	
NH6362	Cs 9	$A_{Ec<1/2\ CTD\text{-}ST>}B_{St}$	52±4%	0.9	-0.031	**<1/2 CTD-ST>** Mean
NH6363	Cs 96	$A_{Ec<1/2\ CTD\text{-}ST>}B_{St}$	65±1%	1.0	-0.034	-0.034±0.004
NH6378	Cs 85	$A_{Ec<CTD\text{-}ST>}\ B_{St}$	77±5%	0.9	-0.037	
NH6379	Cs 71	$A_{Ec<CTD\text{-}ST>}\ B_{St}$	38±10%	1.6	-0.026	
NH6380	Cs 58	$A_{Ec<CTD\text{-}ST>}\ B_{St}$	63±2%	1.1	-0.034	
NH6376	Cs 57.65	$A_{Ec<CTD\text{-}ST>}\ B_{St}$	65±1%	1.1	-0.034	
NH6377	Cs 57.64	$A_{Ec<CTD\text{-}ST>}\ B_{St}$	31±5%	1.0	-0.024	
NH6381	Cs 45	$A_{Ec<CTD\text{-}ST>}\ B_{St}$	63±5%	1.1	-0.034	
NH6382	Cs 33	$A_{Ec<CTD\text{-}ST>}\ B_{St}$	25±11%	1.4	-0.022	
NH6383	Cs 21	$A_{Ec<CTD\text{-}ST>}\ B_{St}$	58±5%	1.1	-0.032	
NH6384	Cs 9	$A_{Ec<CTD\text{-}ST>}\ B_{St}$	37±9%	1.3	-0.026	**<CTD-ST>** Mean
NH6385	Cs 96	$A_{Ec<CTD\text{-}ST>}\ B_{St}$	43±6%	1.5	-0.028	-0.030±0.005
NH6367	Cs 85	$A_{Ec<CTD\text{-}ST>}\ B_{Ec}$	79±5%	0.8	-0.038	
NH6368	Cs 71	$A_{Ec<CTD\text{-}ST>}\ B_{Ec}$	70±4%	0.9	-0.035	
NH6369	Cs 58	$A_{Ec<CTD\text{-}ST>}\ B_{Ec}$	60±16%	1.2	-0.033	
NH6365	Cs 57.65	$A_{Ec<CTD\text{-}ST>}\ B_{Ec}$	68±4%	1.0	-0.035	
NH6366	Cs 57.64	$A_{Ec<CTD\text{-}ST>}\ B_{Ec}$	41±4%	0.8	-0.027	
NH6370	Cs 45	$A_{Ec<CTD\text{-}ST>}\ B_{Ec}$	56±3%	1.2	-0.032	
NH6371	Cs 33	$A_{Ec<CTD\text{-}ST>}\ B_{Ec}$	23±2%	1.5	-0.021	
NH6372	Cs 21	$A_{Ec<CTD\text{-}ST>}\ B_{Ec}$	75±2%	0.9	-0.037	
NH6373	Cs 9	$A_{Ec<CTD\text{-}ST>}\ B_{Ec}$	69±1%	0.7	-0.035	**<CTD-ST>** Mean
NH6374	Cs 96	$A_{Ec<CTD\text{-}ST>}\ B_{Ec}$	85±3%	0.8	-0.039	-0.033±0.005

Table S4. Efficiency of γδ resolution reactions with a supercoil sensor at 4 positions in E. coli strains with a WT and chimeric *Salmonella-E. coli* GyrA fusions.

Strain	GyrA	Sensor Position min/gene	Resolution Efficiency
E. coli NH	*E. coli*	85' *gidB*	94±1%
E. coli NH	*E. coli*	*8' cynX*	87±6%
E. coli NH	*E. coli*	*15' ybfN*	87±4%
E. coli NH	*E. coli*	*59' rrnG*	96±1%
E. coli NH	*S. Tm* <CTD *E. coli*>	85' *gidB*	93±1%
E. coli NH	*S. Tm* <CTD *E. coli*>	*8' cynX*	94±1%
E. coli NH	*S. Tm* <CTD *E. coli*>	*15' ybfN*	96±1%
E. coli NH	*S. Tm* <CTD *E. coli*>	*59' rrnG*	79±4%
E. coli NH	*S. Tm* <32 aa *E. coli* Tail>	85' *gidB*	80±4%
E. coli NH	*S. Tm* <32 aa *E. coli* Tail>	*8' cynX*	77±5%
E. coli NH	*S. Tm* <32 aa *E. coli* Tail>	*15' ybfN*	87±4%
E. coli NH	*S. Tm* <32 aa *E. coli* Tail>	*59' rrnG*	79±4

1. Benjamin, K.R., et al., *Contributions of supercoiling to Tn3 resolvase and phage Mu Gin site-specific recombination.* J. Mol. Biol., 1996. **256**: p. 50-65.
2. Booker, B.M., S. Deng, and N.P. Higgins, *DNA topology of highly transcribed operons in Salmonella enterica serovar Typhimurium.* Mol Microbiol, 2010. **78**(6): p. 1348-64.
